# Adaptive Memory of Human NK-like CD8^+^ T-Cells to Aging, and Viral and Tumor Antigens

**DOI:** 10.3389/fimmu.2016.00616

**Published:** 2016-12-19

**Authors:** María Luisa Pita-López, Alejandra Pera, Rafael Solana

**Affiliations:** ^1^Research Center in Molecular Biology of Chronic Diseases (CIBIMEC), CUSUR University of Guadalajara, Guzmán, Mexico; ^2^Clinical Division, Brighton and Sussex Medical School, University of Sussex, Brighton, UK; ^3^Maimonides Biomedicine Institute of Cordoba (IMIBIC), Reina Sofia Hospital, University of Córdoba, Córdoba, Spain

**Keywords:** NK-like CD8^+^ T-cells, memory, T-cell differentiation, immunosenescence, aging, CMV, natural killer receptors, CD56

## Abstract

Human natural killer (NK)-like CD8^+^ T-cells are singular T-cells that express both T and NK cell markers such as CD56; their frequencies depend on their differentiation and activation during their lifetime. There is evidence of the presence of these innate CD8^+^ T-cells in the human umbilical cord, highlighting the necessity of investigating whether the NK-like CD8^+^ T-cells arise in the early stages of life (gestation). Based on the presence of cell surface markers, these cells have also been referred to as CD8^+^KIR^+^ T-cells, innate CD8^+^ T-cells, CD8^+^CD28^−^KIR^+^ T-cells or NKT-like CD8^+^CD56^+^ cells. However, the functional and co-signaling significance of these NK cell receptors on NK-like CD8^+^ T-cells is less clear. Also, the diverse array of costimulatory and co-inhibitory receptors are spatially and temporally regulated and may have distinct overlapping functions on NK-like CD8^+^ T-cell priming, activation, differentiation, and memory responses associated with different cell phenotypes. Currently, there is no consensus regarding the functional properties and phenotypic characterization of human NK-like CD8^+^ T-cells. Environmental factors, such as aging, autoimmunity, inflammation, viral antigen re-exposure, or the presence of persistent tumor antigens have been shown to allow differentiation (“adaptation”) of the NK-like CD8^+^ T-cells; the elucidation of this differentiation process and a greater understanding of the characteristics of these cells could be important for their eventual in potential therapeutic applications aimed at improving protective immunity. This review will attempt to elucidate an understanding of the characteristics of these cells with the goal toward their eventual use in potential therapeutic applications aimed at improving protective immunity.

## Introduction

T lymphocytes derive their name from their site of maturation in the thymus. In particular, T cytotoxic (Tc) cells that express CD8 are activated upon interaction with an MHC-class I complex on the surface of an altered-self cell (e.g., virus-infected cell or tumor cell) in the presence of appropriate cytokines. T-cell co-signaling is largely context dependent and relies on a diverse array of costimulatory and co-inhibitory receptors spatiotemporally regulated, which may have distinct or overlapping functions in T-cell priming, activation, differentiation, and memory responses (1). The total cytotoxic CD8^+^ T-cell pool is exposed to different microenvironmental stimuli (both TcR dependent and independent) and the resulting phenotype and cytokine secretion will determine an individual T-cell or T-cell clone’s effector or regulating functional capacities, including tissue residence/homing and organ homeostasis (2).

In addition to CD8^+^ T lymphocytes, natural killer (NK) cells have a crucial role in the recognition and killing of virus-infected/tumor cells, but unlike CD8^+^ T-cells, they use a repertoire of germ-line encoded inhibitory/activating receptors that recognize “missing self”/“altered-self” antigens on the target cells leading to cytotoxicity and cytokine production (3). These NK cell receptors (NKRs) are also expressed on certain subsets of T-cells. One example is NKR-CD56, which has been found to be elevated in both peripheral blood cells and in tumor-infiltrating lymphocytes in patients with colorectal cancer (4). In many clinical circumstances, the expression of different NKRs on T-cells is associated with prolonged antigen stimulation, suggesting that these receptors play a crucial role in the homeostasis of antigen-experienced T-cells.

Cumulative evidence supports the existence of T-cell subsets, with characteristics that bridge innate and adaptive immunity, which are relevant in inflammation and viral and tumor surveillance, and which could have a role in the pathogenesis of autoimmune diseases. These NKR-expressing cytotoxic T lymphocytes (CTL) have been termed NK T (NKT) cells. Thus, NKT cells are naturally occurring, although rare, T-cells that express both T and NK cell receptors (5). However, there is some confusion with the use of the term “NKT-cell.” On one hand, CD1d-restricted cells, which have a semi-invariant TcR, are frequently called NKT-cells or invariant NKT (iNKT) cells; on the other hand, highly specialized effector memory CD8^+^ T-cells expressing NKRs are also referred as NKT-like cells. Therefore, to avoid confusion, we will call the later, NK-like CD8^+^ T-cells.

Natural killer-like CD8^+^ T-cell differentiation occurs after the induction of transduction signals that activate/inhibit the expression of certain CD8^+^ T-cells genes, determining the activation state, proliferation, and differentiation (6). Indeed, prolonged antigen stimulation may induce changes in the CD8^+^ T-cell receptor repertoire leading to the expression of NKRs; and chronic antigen stimulation of T cells also leads to other phenotypic changes such as the loss of costimulatory molecules (e.g., CD28) (5). Usually, CD8^+^ T-cell memory subsets display specific responses based on the expression of killer cell immunoglobulin-like receptors (KIRs) used to distinguish unhealthy cellular targets from the healthy host cells (7, 8). However, high antigen concentrations can bypass the KIR-mediated inhibition of T-cell activation. Dynamic KIR expression may mediate T-cell tolerance to self-antigens by down regulating self-reactive T-cells (9). Nevertheless, the functional significance of the inhibitory or activating NKRs on NK-like CD8^+^ T-cells is still unclear.

Natural killer-like CD8^+^ T-cells have been described using different names, for example: CD56^+^CD8^+^ NKT-like cells (10), CD28^−^KIR^+^ CD8^+^ T-cells (5), KIR^+^CD8^+^ T-cells—particularly those expressing NKG2A—(11), or the general term innate CD8^+^ T-cells—since NKR^+^ αβT-cells likely represent immune effector cells that are capable of combining innate and adaptive functions (12). Table 1 summarizes the most relevant information regarding the characterization of NK-like CD8^+^ T-cells.

**Table 1:** **Characterization of NK-like CD8^+^ T-cells**.

Study	Cell name	Specie	Biomarkers	Result	Reference
The expression of NK receptors in PBL from healthy and melanoma patients	CD28^−^ cytolytic effector T cells	Human	CD28^−^ preferential alpha/beta TcR^+^ killer cell immunoglobulin-like receptor (KIR)^+^	The percentages of NK receptor-positive (p58.1, p58.2, p70, p140, ILT2, NKRP1A, ZIN176, CD94, and CD94/NKG2A) T cells (NKT cells) varied more strongly between melanoma patients	Speiser et al. (5)

Changes in the T cell pool caused by CMV infection contribute to immunosenescence	CD8^+^ natural killer (NK) T-like cells	Human	CD56^+^	NKT-like cell percentage increases with the combination of both CMV and age	Hassouneh et al. (10)

Eomesodermin-expressing innate-like CD8^+^ KIR/NKG2A^+^ T cells in human adults	KIR/NKG2A^+^ CD8^+^ T cells	Human	KIR^+^ (NKG2A) innate/memory phenotype	Increased Eomes expression, prompt IFN-gamma production in response to innate-like stimulation by IL-12 + IL-18	Jacomet et al. (11)

Review: expansions of NK-like alpha/beta TcR T cells with chronologic aging	NK-like alpha/beta TcR T cells	Human	NK cell receptors on aged alpha/beta TcR cells	NKR expression on T cells is physiologically programed rather than a random event of the aging process	Vallejo et al. (12)

Regulation of the immune response through killing antigen-bearing DCs	CD8^+^ NKT-like cells	Mice	TcR beta CD3 NK1.1^+^ CD49b^+^ NKG2D^+^	Secretion of high levels of IFN-gamma, but not IL-4	Wang et al. (13)

T-cell responses and protective immunity	Memory-like effector NKT cells	Mice	CD44^+^ CD62L^−^	Coadministration of alpha-GalCer analog and TLR4 agonist activates memory-like effector NKT cells	Coelho-Dos-Reis et al. (14)

**Figure 1:**
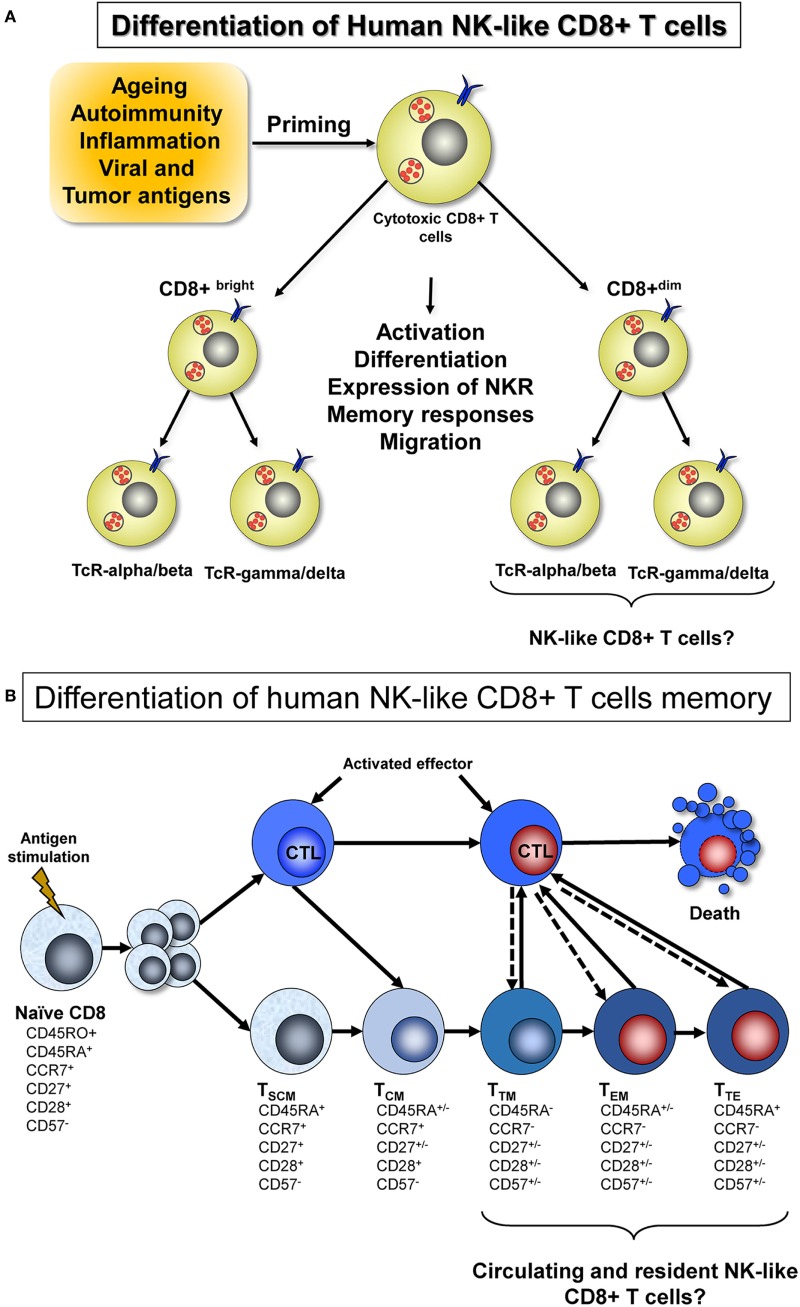
**(A)** Differentiation of human NK-like CD8^+^ T-cells and expression of TcR. The total pool of cytotoxic CD8^+^ T-cells is exposed to different TcR-alpha/beta and TcR-gamma/delta dependent or independent microenvironmental stimuli. From this pool originate the NK-like CD8^+^ T cells, which can be induced by the transduction of signals that activate or inhibit gene expression that, in turn, determines cytokine secretion, effector/regulating functions, migration/tissue retention, activation state, proliferation, and differentiation. Other factors that influence this process are aging, autoimmunity, inflammation, and the presence of viral and tumor antigens. **(B)** Differentiation of human NK-like CD8^+^ T-cell memory. Differentiation of CD8^+^ T-cell memory starts after naïve T-cell activation. According to the recently proposed model of progressive differentiation, the fate of T-cells depend on the duration of signaling and the presence or absence of cytokines (17). Thus, a single naïve cytotoxic T lymphocyte will differentiate gradually to different memory subsets: stem cell memory (T_SCM_), central memory (T_CM_), transitional memory (T_TM_) cells, effector memory (T_EM_), and terminally differentiated effector memory (T_TE_). In consequence, brief antigen stimulation will generate T_CM_ cells and T_TM_ cells, the later being more differentiated than T_CM_ cells but not as fully differentiated as T_EM_ cells, in terms of phenotype. On the other hand, sustained stimulation together with presence of cytokines will generate T_EM_ and T_TE_ cells, which most probably include NK-like CD8^+^ T cells both circulating or tissue resident.

In both the human umbilical cord and in healthy adults, an “innate/memory-like” CD8^+^ T-cell subset that expresses KIR and NKG2A has been described. These cells were EOMES^hi^, exhibited potent antigen-independent cytotoxic activity, and produced IFN-gamma in response to IL-12 + IL-18 (11). The differentiation of these cells, similar to that of the overall pool of CD8^+^ T-cells, can be influenced by aging. Specifically, age has been associated with increased susceptibility to infections and inflammatory diseases (15), cancer, and autoimmunity (16). The differentiation of human NK-like CD8^+^ T-cells is initiated after viral/tumor antigen priming and may be influenced by other factors such as aging, autoimmunity, or inflammation. These cytotoxic CD8^+^ T-cells, when activated, implement a differential expression of NKRs, leading to memory and migration responses. According to the expression of the CD8 marker on their surface, these cells are classified as bright or dim and display different functional properties (Figure 1A). In this context, we reviewed the literature regarding NK-like CD8^+^ T-cells and their phenotypic characterization associated with viral infection, immunosenescence, and diseases.

## Phenotypic Characterization of NK-like CD8^+^ T-Cells

Immunological memory is the ability of the immune system to respond more rapidly and effectively to previously encountered pathogens. This is a classical feature of adaptive immunity, which is derived from unique patterns of gene expression. A faster and stronger transcription of previously activated genes occurs in memory T-cells compared with naïve cells. This ability to remember past transcriptional responses is termed “adaptive transcriptional memory” (18). After acute infections, CD8^+^ T-cell memory differentiation leads to the generation of functionally distinct populations, with either proliferative potential or cytotoxic effector functions, that recirculate into lymphoid tissues or remain tissue-resident (6). This phenomenon depends on the expression of several receptors, including the C-C chemokine receptor 7 (CCR7) and CD45RA, which have been used to discriminate naïve (*N*; CD45RA^+^CCR7^+^), central memory (T_CM_; CD45RA^−^CCR7^+^), transitional memory (T_TM_; CD45RA^−^CCR7^−^), and terminally differentiated T-cells (T_EMRA_ or T_TE_: CD45RA^+^CCR7^−^) (15). A further classification divides the CCR7^−^CD8^+^ T-cell subpopulation into three distinct memory subsets according to the expression of CD45RA: CD45RA^null^, CD45RA^dim^, and CD45RA^bright^ (19).

There are several models of memory CD8^+^ T-cell differentiation (20) and two new subsets have been recently described: the “stem cell-like memory T-cells” (T_SCM_) (21) and the “transitional memory (T_TM_) T-cells” (17). Differentiation of circulating memory CD8^+^ T-cells starts after antigen challenge and subsequent naïve T-cell activation. The recently described progressive differentiation model proposes that the fate of the T-cells depends on the duration of signaling and the presence or absence of cytokines. Thus, a single naïve cytotoxic T lymphocyte will differentiate gradually to different memory subsets (Figure 1B). In consequence, brief antigen stimulation will generate T_CM_ and T_TM_ cells, while sustained stimulation together with the presence of cytokines will lead to T_EM_ and T_TE_ (T_EMRA_) cells that re-express CD45RA (17, 22–24). Additionally, another type of memory cell has been described, the “resident memory T-cells” (T_RM_), which are non-recirculating memory T-cells with long-term persistence in epithelial barrier tissues. As shown in Figure 1B, it is probable that NK-like CD8^+^ T-cells emerge from T_TM_, T_EM_, or T_TE_ CD8^+^ T-cell phenotypes; these cells could be circulating or tissue resident.

In addition, some authors suggest that the CD45RO^+^CD45RA^−^ T-cells comprise diverse memory subsets, including T_CM_, T_SCM_, T_EM_, and T_RM_ subsets, which are heterogeneous in their generation, distribution, and function (24). Thus, T_RM_ cells may persist in the absence of antigens and display several effector functions. Moreover, T_RM_ cells could have evolved to provide rapid immune protection against pathogens. However, autoreactive, aberrantly activated, and malignant T_RM_ cells can contribute to numerous human inflammatory diseases (25).

CD8^+^ T-cells in lymphoid tissues are naïve, while in mucosal sites, these cells are IFN-gamma producing T_EM_ cells. The T-cell activation marker, CD69, is constitutively expressed by memory T-cells in all tissues, distinguishing them from circulating subsets. T_RM_ cells expressing CD69 are also present in human mucosal and peripheral tissue sites (24). However, the mucosal memory T-cells exhibit additional distinct phenotypic and functional properties (26). In particular, human intrahepatic lymphocytes are rich in CD1d-unrestricted T-cells that co-express NKRs (NK-like CD8^+^ T-cells), and it is possible that the hepatic epithelial cells and the cytokine milieu play a role in the shaping of these cells. For example, IL-15 is capable of inducing Ag-independent upregulation of NKRs in the CD8^+^CD56^−^ T-cells. This increased percentage of intrahepatic NK-like CD8^+^ T-cells could be in part due to a local CD8^+^ T-cell differentiation (27) and could explain how NK-like CD8^+^ T-cells differentiate in the human liver.

The distribution and tissue residence of naïve, central and effector memory, and terminal effector subsets is contingent on both their differentiation state and tissue localization. Moreover, T-cells homeostasis, driven by cytokine or TcR-mediated signals, is different in CD4^+^ or CD8^+^ T-cell lineages and varies with their differentiation stage and tissue localization (28). In this sense, it is important to investigate NK-like CD8^+^ T-cells with respect to memory phenotype, functional properties, and long-term differential fates following acute infection or chronic diseases.

## NK-like CD8^+^ T-Cells in Virus Infection and Immunosenescence

Throughout an individual’s lifetime, the memory T-cell percentage undergoes dynamic changes that can be classified into three phases: memory generation during infancy and early childhood, memory homeostasis, which occurs after age 20–25, and immunosenescence (24). This last term refers to the deterioration of the immune system associated with aging, and it is characterized by substantial alterations of the T-lymphocyte subsets (29). An increased expression of NK cell markers on T-cells has been reported to be associated with aging and chronic activation of the immune system, as reflected in the accumulation of effector/senescent T-cells (30). This memory subpopulation is interesting, because the senescence of human T_TE_ CD8^+^ T-cells is stringently controlled by distinct and reversible cell signaling events (31). Also, there is evidence of a differential regulation of NKR expression between T-cells and NK cells suggesting that NKR expression on T-cells is physiologically programed rather than a random event of the aging process (12). This may suggest that the NK-like CD8^+^ T-cells have a functional plasticity with respect to their “adaptation” that allows them to respond to different stimuli.

CMV and HIV infection strongly affect CD8^+^ T-cell differentiation and maturation, enhancing immunosenescence due to the accumulation of highly differentiated T_EM_ and T_TE_ cells (32, 33). The CD57 antigen has been traditionally used to characterize terminally differentiated “senescent” cells, as CD57^+^ T-cells exhibit a reduced proliferative capacity and altered functional properties (34). Of note, the expansion of CD57^+^CD8^+^ T-cells is a hallmark of latent CMV infection (35). The CD57^+^CD8^+^ T-cell subset is functionally heterogeneous, and includes highly cytotoxic T_TE_ cells that express intermediate levels of EOMES, as well as non-cytotoxic EOMES^hi^ T_TE_ cells with high proliferative capacity (36). These above-referenced studies highlight the existence of functional heterogeneity among the CD8^+^ T-cell memory subsets. Regarding NK-like CD8^+^ T cells, high percentages of these cells also express the CD57 marker and likewise arise after CMV infection (10). It has been well established that NK-like CD8^+^ T-cells expand with age (30, 37–39) and some studies suggest that their frequency is increased in CMV-seropositive individuals (40–42). However, recent work performed in healthy young individuals indicates that NK-like CD8^+^ T-cell frequency is not affected by CMV latent infection. Thus, the authors propose that these cells accumulate with age in the CMV-seropositive individuals, rather than with CMV infection *per se* (10).

Moreover, NK-like CD8^+^ T-cells from Epstein–Barr virus (EBV)-associated tumor patients are quantitatively and functionally impaired and in a human-thymus-SCID chimera model, the EBV-induced human NK-like CD8^+^ T-cells synergize with NK-like CD4^+^ T-cells suppressing EBV-associated tumors upon induction of a Th1-bias (43). Additionally, in women with human papillomavirus (HPV)-associated cervical neoplasia, there are increased levels of CD28^−^, T_EM_, and CD16^+^CD56^+^ CD8^+^ T-cells in peripheral blood, probably associated with the chronic infection with HPV (44). As we mentioned above, NK-like CD8^+^ T-cells possess a diverse TcR repertoire and there is evidence that these cells can function as antigen-specific suppressive cells that regulate the immune response through killing antigen-bearing dendritic cells (13). The class-I MHC-restricted T-cell-associated molecule (CRTAM) has been shown to be expressed only on activated class-I MHC-restricted T-cells, including NK-like CD8^+^ and conventional CD8^+^ T-cells. Of note, this molecule is a surface marker of activation associated with human viral infections and autoimmune diseases (45). These studies show that the NK-like CD8^+^ T-cells interact with other cells and that chronic stimulation determines their phenotype.

## NK-like CD8^+^ T-Cells and Disease

There is evidence in the literature of an immune suppressor role for the CD8^+^CD28^−^ T-cells (Ts) and the CD3^+^CD56^+^ T-cells. Patients with B-cell non-Hodgkin’s lymphoma had significantly higher percentages of Ts cells and NKT-like cells than healthy people, suggesting that, in this type of lymphoma, these cell subsets may possibly have an immunosuppressive role (46). It has been suggested that tumor-induced dysfunction of CTL in patients with multiple myeloma may contribute to immune escape and causes clonal T-cell immunosenescence, but not exhaustion, as a predominant feature. These cells exhibited a senescent secretory effector phenotype: KLRG-1^+^/CD57^+^/CD160^+^/CD28^−^ (47) and may possibly be NK-like CD8^+^ T-cells with T_EM_ or T_TE_ phenotype. Furthermore, the use of *ex vivo*-expanded NK and NK-like T-cells has been reported seems to be safe and it could be an approach for further clinical evaluation in cancer patients (47).

Patients with Behcet’s uveitis also showed an increased number of CD8^+^ T-cells and CD8^+^CD56^+^ (NKT-like) cells in the aqueous humor, indicating a possible role for these subsets in the immunopathogenesis of the disease (48). CD56^+^CD8^+^ NKT-cells express more IFN-gamma and KIR in patients with leishmaniasis compared with healthy subjects (49). Similarly, loss of CD28 was associated with an increased percentage of T and NK-like T-cells producing IFN-gamma or TNF-alpha in patients with chronic obstructive pulmonary diseases (44). Furthermore, targeting peripheral blood pro-inflammatory CD28^−^ T-cells and NK-like CD8^+^ T-cells by inhibiting CD137 expression may possibly be of relevance to the treatment of bronchiolitis obliterans syndrome (50). In this regard, the percentage of CD57^+^CD8^+^ T-cells is the strongest immunologic predictor of future cutaneous squamous cell carcinoma and was correlated with increasing CD8^+^ T-cell differentiation (36). As mentioned above, a high percentage of CD57^+^CD8^+^ T cells are NK-like.

The human activating receptor NKG2D recognizes a diverse family of ligands (MICA, MICB, and ULBPs 1–6), leading to the activation of effector cells and triggering the lysis of target T-cells. Differential expression of NKG2D is regulated in the different T-cell subsets by epigenetic mechanisms (51). The NKG2D receptor–ligand system plays an important role in the immune response to infections, tumors, transplanted grafts, and autoantigens. In lung cancer patients, NK-like CD8^+^ T-cells exhibit low expression of NKG2D, which correlates with the pathological stage (52). Thus, understanding the regulation of human NK-like CD8^+^ T-cells activation could be a strategy to manipulate T-cell-mediated responses including tumoral responses and infections.

Patients with Behcet’s uveitis also showed an increased number of CD8^+^ T-cells and CD8^+^CD56^+^ (NKT-like) cells in the aqueous humor, indicating a possible role for these subsets in the immunopathogenesis of the disease (48). A skewed distribution and lower frequencies of circulating activated CD161^+^ NK-like CD8^+^ T-cells was observed in patients with common variable immunodeficiency disorders, suggesting a probable regulatory function of these cells (53). CD161 is expressed by several T-cell subsets, including CD8^+^, NK-like CD8^+^, CD4^+^, and TcR-gamma/delta cells and all CD161^+^ lymphocytes display a shared innate response to IL-12 + IL-18 in which CD161 can act as a costimulatory receptor (54). Additionally, IL-23 responsiveness is restricted to the CD161^+^ subset in CD45RO^+^CD8^+^ memory T-cells (55). Moreover, both the frequency and the absolute number of CD161^+^CD8^+^ T-cells are decreased in the peripheral blood of patients suffering from systemic lupus erythematosus (56). A skewed distribution and lower frequencies of circulating activated CD161^+^ NK-like CD8^+^ T-cells was observed in patients with common variable immunodeficiency disorders, suggesting a probable regulatory function of these cells (53).

Finally, evidence from murine research has shown that harnessing the immune adjuvant properties of NK-like CD8^+^ T-cells can be an effective strategy to generate immunological memory and anticancer immunity. This effect was associated with the IFN-gamma-dependent expansion of KLRG1^+^CD8^+^ effector T-cells (57). Another study in mice assessed the vaccine induction of CD8^+^ T-cell responses and protective immunity after coadministration of alpha-GalCer analog and TLR4 agonist. The results showed a robust CD8^+^ T-cell response to PyCS protein and WT-1 antigen and activation of memory-like effector NK-like CD8^+^ T-cells, with a CD44^+^CD62L^−^ phenotype (14).

## Future Research Considerations

This review supports the concept that NK-like CD8^+^ T-cells are part of a cell subset associated with the acquisition of differential marker profiles, although there is little information regarding the functional properties of these cells in humans. Thus, there are several questions to clarify. First, are NK-like CD8^+^ T-cells CD3_+_/CD8^+^/CD56^+^ bright or dim? Second, are they TcR-alpha/beta^+^, TcR-gamma/delta^+^, or TcR-gamma/delta^−^? Third, do these cells contain variant or semi-invariant chains? Fourth, do they have CD8^+^/alpha-beta or CD8^+^/alpha-alpha? It would also be interesting to evaluate whether these subpopulations differ in their abilities to stimulate other immune cells and if they have diverse immunoregulatory functions including activation/suppression of diverse cells. Additionally, more information is needed with regards to how different environmental factors, such as autoimmunity, inflammation, viral antigen re-exposure, or persistent tumor antigens might allow the differentiation (“adaptation”) of the memory NK-like CD8^+^ T-cells. Finally, it is important to be cognizant of the different NK/T-cell-like cell populations and exclude the NK-like CD8^+^ T-cells when analyzing the immune responses mediated by conventional CD8^+^ T-cells and vice versa.

Nutrient/metabolic regulators can influence NK-like CD8^+^ T-cell differentiation, which could be analyzed through nutrigenomics contributing to the knowledge regarding the differentiation of this subset. As the balance between activating/inhibitory receptors controls NK-like CD8^+^ T-cells immune responses, it should also be considered that the expression of these receptors could depend on the cell differentiation state, the age, and/or diseases of the individual.

In conclusion, a thorough functional and phenotypic characterization of human NK-like CD8^+^ T-cells will be fundamental in order to provide mechanistic insight into the functional adaptation of these cells to aging, autoimmunity, inflammation, viral, and tumor antigens and toward their exploitation in potential therapeutic applications.

## Author Contributions

M-PL conceived and participated in the design and coordination of the manuscript. AP and RS provided helpful discussions and edited the manuscript. All authors wrote, read, and approved the final manuscript.

## Conflict of Interest Statement

The authors declare that the research was conducted in the absence of any commercial or financial relationships that could be construed as a potential conflict of interest.
